# Are Deep Tissue Cultures a Reliable Alternative to Bone Biopsy for Diagnosing Diabetic Foot Osteomyelitis? A Comparative Diagnostic Study

**DOI:** 10.3390/diagnostics15070880

**Published:** 2025-04-01

**Authors:** Serap Ulusoy, İbrahim Kılınç, Belgin Coşkun, Müge Ayhan

**Affiliations:** 1 General Surgery, Ankara Bilkent City Hospital, Çankaya 06800, Turkey; ikilinc8083@gmail.com; 2 Infectious Diseases and Clinical Microbiology, Ankara Bilkent City Hospital, Çankaya 06800, Turkey; belgintekin@yahoo.com; 3 Infectious Diseases and Clinical Microbiology, Ankara Yıldırım Beyazıt University, Ankara Bilkent City Hospital, Çankaya 06800, Turkey; dr.mugeayhan@hotmail.com

**Keywords:** diabetic foot osteomyelitis, deep tissue culture, bone biopsy, microbiology, diabetic foot infections, *Staphylococcus aureus*, diagnostic accuracy

## Abstract

**Background:** Diabetic foot osteomyelitis (DFO) is a serious complication of diabetic foot ulcers (DFUs) that contributes to high morbidity and an increased risk of lower extremity amputation. While bone biopsy cultures are considered the gold standard for identifying causative pathogens, their invasive nature limits widespread clinical use. This study evaluates the microbiological concordance between deep tissue and bone cultures in diagnosing DFO. **Methods:** A retrospective analysis was conducted on 107 patients with DFO who underwent simultaneous deep tissue and bone biopsy cultures. Patient demographics, ulcer classification, and microbiological culture results were recorded. The agreement between deep tissue and bone cultures was assessed to determine the diagnostic utility of deep tissue sampling. **Results:** The overall concordance between deep tissue and bone cultures was 51.8%. *Staphylococcus aureus* was the most frequently isolated pathogen in both culture types and had the highest agreement rate (44.4%). Concordance rates were lower for Gram-negative bacteria (31.9%) and other Gram-positive microorganisms (24.2%). In 21.2% of the cases, pathogens were isolated only from deep tissue cultures, while 16.5% had positive bone cultures but negative deep tissue cultures. **Conclusions:** Deep tissue cultures demonstrate moderate microbiological concordance with bone biopsy in the diagnosis of DFO, particularly in cases with monomicrobial *Staphylococcus aureus* infection. While bone biopsy remains the gold standard, deep tissue cultures may be a practical alternative when bone sampling is not feasible or for patients unsuitable for surgery. However, their limited reliability in detecting Gram-negative and polymicrobial infections underscores the need for more accurate, less invasive diagnostic tools. Future research should focus on validating molecular and advanced diagnostic methods to improve clinical decision-making and patient outcomes in DFO.

## 1. Introduction

Diabetes mellitus (DM) is a metabolic disorder that has become an increasing global health burden and is now recognized as one of the most significant public health challenges worldwide. Current epidemiological data estimate that approximately 828 million individuals worldwide are living with diabetes, reflecting an increase of 630 million cases since 1990 [1]. This dramatic rise in diabetes prevalence has resulted in a parallel increase in diabetes-related complications, thereby placing an escalating burden on healthcare systems.

DM is associated with a wide range of complications that contribute to increased morbidity and mortality, with diabetic foot ulcers (DFUs) representing a particularly significant clinical challenge due to their impact on the lower extremities [2]. It is estimated that 19–34% of individuals with diabetes will develop a DFU at some point in their lifetime, with up to 60% of these ulcers becoming complicated by infection. The severity of these infections varies, and it is estimated that 20–60% of diabetic foot infections involve the underlying bone, leading to diabetic foot osteomyelitis (DFO) [3,4,5]. DFO is a serious and complex complication associated with an increased risk of amputation, prolonged antibiotic therapy, delayed wound healing, high recurrence rates, and extended hospitalization periods, making its accurate diagnosis and appropriate management crucial for improving patient outcomes [4,5]. DFO can be treated with antibiotic therapy, surgical debridement, amputation, and, more recently, minimally invasive surgical techniques developed to prevent limb loss [6]. However, there is currently no clear consensus in the literature regarding the superiority of one treatment approach over another. Therefore, regardless of the therapeutic modality applied, one of the most critical determinants of treatment success is accurate and timely diagnosis.

The presence or absence of osteomyelitis in DFU is a critical determinant of ulcer prognosis. Accurate diagnosis of osteomyelitis is essential for optimizing treatment strategies, preventing unnecessary and prolonged antibiotic use, and minimizing the need for invasive surgical interventions [7]. Early and definitive diagnosis is essential to avoid inappropriate therapeutic decisions and prevent disease progression. However, the absence of universally accepted diagnostic criteria and the variability among currently available diagnostic tests complicate the clinical decision-making process [8,9]. Although a combination of clinical assessment, radiological imaging, and laboratory testing is considered to enhance diagnostic accuracy, the existing evidence highlights the limitations of each method, and the most effective diagnostic approach remains uncertain [10]. Moreover, emerging molecular diagnostic techniques and advanced imaging modalities, such as positron emission tomography–computed tomography (PET-CT) and magnetic resonance imaging (MRI), have been proposed to improve diagnostic precision, but their cost, availability, and standardization remain challenging in routine clinical settings [11].

The microbiological and histopathological analysis of bone biopsy specimens is considered the gold standard for diagnosing osteomyelitis, enabling the precise identification of causative pathogens and their antibiotic susceptibility profiles [12,13]. However, bone biopsy may not always be feasible in routine clinical practice due to several limitations, including the requirement for an experienced clinical team, specialized equipment, and the potential risk of procedural complications, such as fractures or the contamination of adjacent tissues. Additionally, disparities in accessibility to bone biopsy procedures across different healthcare settings and geographical regions further complicate its widespread adoption [14].

Although international guidelines recommend tailoring antimicrobial therapy based on bone biopsy culture results, real-world clinical practice often relies on soft tissue culture results to guide treatment decisions. Given the potential spread of infection from superficial soft tissue to bone in DFO, deep tissue cultures obtained from the ulcer base have been proposed as an alternative diagnostic tool. However, their ability to reliably reflect bone infection remains controversial [15]

In this study, we aim to evaluate whether deep tissue cultures serve as a reliable alternative to bone biopsy cultures in the diagnosis of DFO. By analyzing the microbiological concordance between deep tissue and bone biopsy cultures, we seek to contribute to the development of more effective and practical diagnostic strategies for improving DFO management and treatment approaches. Additionally, we aim to explore the clinical implications of adopting deep tissue cultures as a diagnostic tool and their potential role in guiding targeted antibiotic therapy, thereby optimizing patient outcomes while addressing the limitations associated with bone biopsy procedures [16]. Accordingly, we hypothesize that deep tissue cultures, due to their ease of application, lower complication risk, and widespread feasibility even in resource-limited settings, may serve as a practical and accessible alternative to bone biopsy cultures for the diagnosis of DFO.

## 2. Materials and Methods

### 2.1. Ethical Approval

This study was approved by the Ethics Committee of Ankara Bilkent City Hospital (TABED 1-24-820). All participants were provided with detailed information regarding the study’s purpose and design, and both verbal and written informed consent were obtained. All study procedures were conducted in accordance with the ethical standards of the institutional review board and the principles of the 1964 Declaration of Helsinki and its subsequent revisions.

### 2.2. Study Population

This retrospective study analyzed patients who were followed up in the Chronic Wound Unit of our hospital between 1 January 2024 and 1 July 2024 due to diabetic foot wounds.

The Chronic Wound Unit operates with a multidisciplinary approach and serves as a tertiary referral center, evaluating and treating approximately 400 diabetic foot patients per month.

Patients who met the eligibility criteria were retrospectively evaluated, and all data were obtained from the hospital’s electronic medical record system.

**Inclusion criteria:**

Patients were included if they fulfilled all of the following conditions:

- Clinical diagnosis of diabetic foot infection (DFI);
- Positive probe-to-bone (PTB) test (defined as palpable bone through the ulcer using a sterile blunt metal probe);
- Radiographic findings consistent with osteomyelitis on initial or follow-up X-rays;
- Underwent surgical intervention (debridement or amputation) during which deep tissue and bone culture samples were intended to be collected simultaneously;
- If bone culture could not be obtained intraoperatively due to technical limitations, patients were still included provided that osteomyelitis was supported by both clinical and radiological evidence;
- Age ≥ 18 years;
- No antibiotic therapy at the time of culture sampling.

**Exclusion criteria:**

Patients were excluded if they met any of the following conditions:

- Clinical or radiological findings consistent with Charcot neuroarthropathy;
- Non-diabetic causes of osteomyelitis (e.g., trauma, malignancy);
- Presence of orthopedic implants or foreign bodies in the affected foot;
- Did not undergo surgical intervention and therefore had no intraoperative culture sample;
- Negative PTB test and/or no radiographic evidence of osteomyelitis;
- Incomplete clinical or microbiological data.

**Figure 1:**
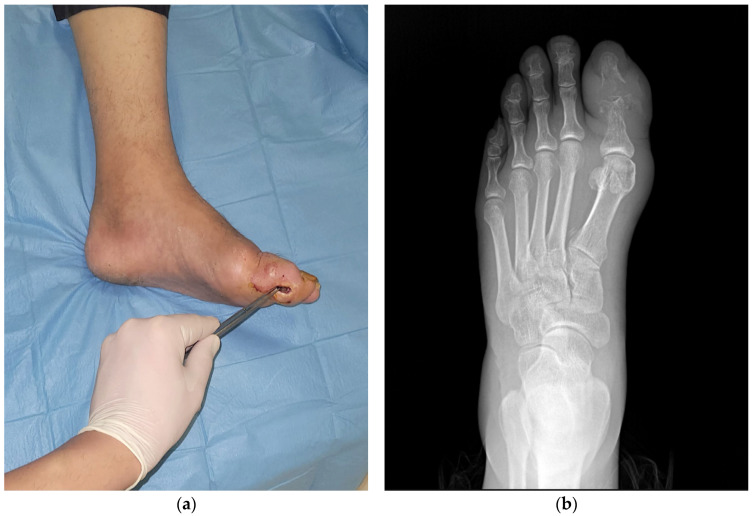
(**a**): Clinical image of a 68-year-old male patient with diabetic toe osteomyelitis. IDSA/IWGDF ulcer classification: Grade 3. The ulcer shows a positive probe-to-bone test. (**b**): Corresponding X-ray image of the same patient demonstrating findings consistent with osteomyelitis. Cortical irregularities, osteolytic lesions, and soft tissue edema are visible at the level of the interphalangeal joints.

**Figure 2:**
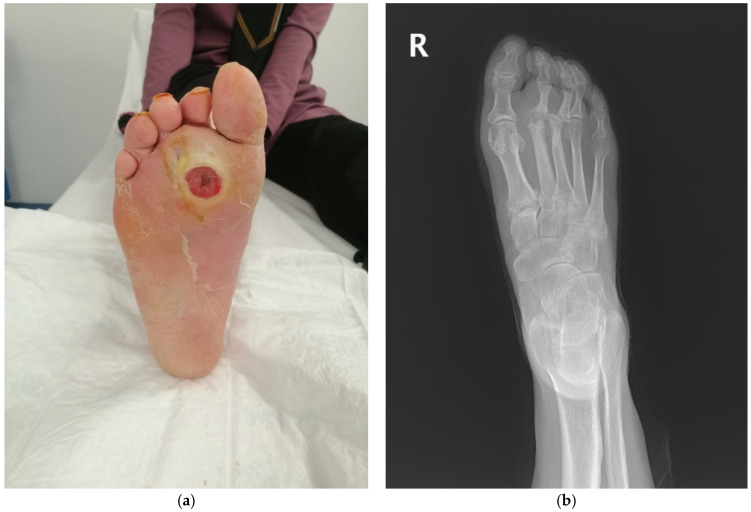
(**a**): Clinical image of a 62-year-old female patient with a neuropathic diabetic foot ulcer located on the plantar surface. IDSA/IWGDF ulcer classification: Grade 3. (**b**): Corresponding X-ray image of the same patient showing radiographic findings consistent with osteomyelitis. Osteolytic lesions, cortical erosion, and irregularities are observed in the phalanges and metatarsal bones.

Representative clinical and radiological images from two patients included in this study are shown in Figure 1a,b and Figure 2a,b. These examples illustrate the typical presentation of DFO, including positive test results and radiographic signs, such as cortical disruption, osteolysis, and soft tissue swelling.

The demographic characteristics of the patients were recorded, including age, sex, comorbidities, prior antibiotic use, and classification of the ulcer according to the Infectious Diseases Society of America/International Working Group of the Diabetic Foot (IDSA/IWGDF) classification system [17], along with the causative microorganisms isolated from deep tissue and bone cultures.

All data were obtained from the hospital’s electronic medical record system.

### 2.3. Specimen Collection

The IDSA/IWGDF classification system [17] was used to grade the DFUs. Simultaneous deep tissue and bone samples were collected intraoperatively under peripheral nerve blockade by the same team. Following the removal of necrotic tissues and superficial debris from the ulcer base, deep tissue biopsy specimens measuring approximately 4–5 mm in diameter were obtained under aseptic conditions using a sterile scalpel. Bone samples were collected either during conservative surgical debridement or amputation, depending on patient-specific factors, such as the extent and severity of infection, the condition of the affected bone, the presence of necrosis or gangrene, clinical urgency, and the patient’s preference regarding limb preservation. In cases where limb-sparing surgery was feasible, debridement was performed and bone specimens were obtained from visibly infected or necrotic areas. In more advanced or non-salvageable cases, amputation was performed to achieve infection source control, and bone samples were collected during the procedure. Throughout the sampling process, strict aseptic conditions were maintained to minimize the contamination risk. To prevent cross-contamination, surgical instruments and gloves were changed as necessary and potential contamination sources were eliminated. Standard culturing techniques were used and all conditions were similar for all patient samples. Our hospital has an in-house microbiology laboratory. All the collected specimens were placed into a transport medium and sent to our hospital microbiology laboratory for culturing within 30 min of data collection.

### 2.4. Microbiological Analysis

Each sample was inoculated onto both 5% sheep blood agar and MacConkey agar plates. All cultures were incubated under aerobic conditions at 35–37 °C for 48 h. Following visible microbial growth, Gram staining was performed directly from the colonies on the culture plates. The identification of isolates in tissue and bone specimen cultures and antibiotic susceptibility tests were performed with a VITEK-2 (bioMérieux, Marcy-I’Ètoile, France) automated identification device. Methicillin resistance in *Staphylococcus* isolates was evaluated with cefoxitin. Resistance rates and MIC (minimal inhibitory concentration) values were determined according to EUCAST (the European Committee on Antimicrobial Susceptibility Testing) standards [18]. Anaerobic bacteria were not included in this study because anaerobic cultures were not performed in our laboratory.

### 2.5. Statistical Analysis

Descriptive statistical analysis was performed using IBM SPSS Statistics 25 software (SPSS Inc., Chicago, IL, USA, 2011). Age is expressed as a mean ± standard deviation. Sex, frequency of comorbid diseases and situations, grade of ulcers, identified microorganisms, and concordance between deep tissue and bone sample cultures are expressed as numbers and percentages.

## 3. Results

**Figure 3:**
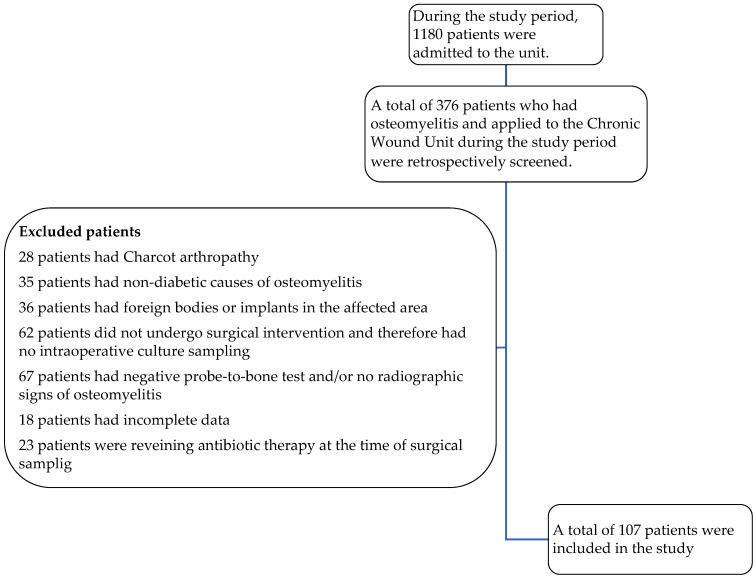
Flowchart of inclusion and exclusion of patients.

As shown in Figure 3, a total of 107 patients were included in this study. The mean age was 63.4 ± 12.3 years, and 74.8% (*n* = 80) of the participants were male. All the patients had at least one comorbidity. The most common comorbid conditions included hypertension (93.5%), peripheral neuropathy (91.5%), peripheral vascular obstruction (89.7%), and atherosclerosis (75.7%) Prior exposure to antibiotics was noted in 96.3% of patients (*n* = 103) (Table 1).

All patients had been off antibiotics for at least 15 days at the time of hospital admission. However, 96.3% had a documented history of systemic antibiotic therapy within the three months prior to admission. The most frequently used agents included β-lactam/β-lactamase inhibitor combinations, cephalosporins, and fluoroquinolones. The duration of prior antibiotic use ranged from 5 to 56 days.

According to the IDSA/IWGDF ulcer classification system, grade 3 ulcers were the most prevalent (64.5%), followed by grade 4 (30.8%) and grade 2 (4.7%) ulcers (Table 2).

Deep tissue cultures were available for 107 patients, and bone cultures were available for 105 of the patients. All infections were monomicrobial with a dominant pathogen identified in every culture that showed microbial growth. In total, 85 patients had microorganism growth in any cultures.

The most identified pathogen in both the deep tissue and bone cultures was *Staphylococcus aureus* (*S. aureus*). It was isolated from 13.1% of the patients (Table 3). The second most common pathogen in deep tissue cultures was *Escherichia coli* (*E. coli*), while the second most common agent in bone cultures was *Klebsiella pneumonia* (*K. pneumonia*).

While the same pathogen was isolated from both the deep tissue and bone cultures in 51.8% of the patients, by contrast, in 10.6% of cases, there was a discordance between the pathogens isolated from deep tissue and bone samples. In 21.2% of patients, bacterial growth was detected only in the deep tissue culture, while no growth was observed in the bone culture. Similarly, in 16.5% of patients, growth was observed only in the bone culture, whereas no pathogen was detected in the deep tissue culture (Table 4).

As mentioned above, 51.8% cases had concordance between the results from the deep tissue and bone cultures. When the concordance was evaluated according to the identified pathogen, the highest concordance between the deep tissue and bone cultures was in patients where *S. aureus* (44.4%) was identified, followed by Gram-negative (31.9%) and then other Gram-positive microorganisms (24.2%) (Table 5).

To further quantify the agreement between the two methods, Cohen’s kappa coefficient was calculated. The value was 0.48, indicating moderate agreement.

## 4. Discussion

This study aims to evaluate the microbiological concordance between deep tissue and bone biopsy cultures in the diagnosis of DFO, and to analyze the diagnostic accuracy of deep tissue cultures. The accurate microbiological diagnosis of DFO is crucial for determining the appropriate treatment protocols.

In our study, the overall concordance rate between deep tissue and bone biopsy cultures was found to be 51.8%. This finding indicates that, in one out of every two patients, the same pathogen was isolated in both culture types. The highest concordance rate was observed in patients with *S. aureus* isolation (44.4%), while lower concordance rates were found in Gram-negative bacteria (31.9%) and other Gram-positive microorganisms (24.2%). These findings suggest that, although deep tissue cultures have a limited but still meaningful diagnostic value compared to bone biopsy, their limitations should be considered, particularly in microorganisms with lower concordance rates. The use of deep tissue cultures alone may not always be sufficient for an accurate diagnosis of osteomyelitis.

DFIs are a common health problem in individuals with diabetes and often lead to serious complications, such as amputation. These infections typically originate from an open wound in the skin and soft tissue but, in most cases, progress to involve the underlying bone, resulting in osteomyelitis. DFO is an infection that develops as a consequence of long-standing diabetes, and is frequently associated with advanced peripheral neuropathy, peripheral arterial disease (PAD), foot deformities, and inadequate foot care [19,20].

In our study, peripheral neuropathy and PAD were observed in 91.5% and 89.7% of patients, respectively—both of which are well-established risk factors for DFO. These rates are considerably higher than those reported in the literature. For instance, the Eurodiale study by Prompers et al. found PAD in 49% of patients with DFUs across Europe [21]. Similarly, recent international guidelines on the management of DFUs report PAD prevalence ranging from 40% to 60% in this population [22]. Regarding peripheral neuropathy, the literature suggests prevalence estimates between 50% and 75%, depending on patient characteristics and diagnostic criteria [23,24]. The higher prevalence observed in our cohort may be explained by the fact that our center functions as a national referral facility for advanced-stage diabetic foot cases. Many of these patients present with severe ischemia, extensive ulceration, and long-standing neuropathic complications. In particular, the majority of admitted patients had stage three or four ulcers, which contributed to the increased severity of infection and ischemic burden.

The management of DFO often involves surgical debridement, long-term antibiotic therapy, or a combination of both [25,26,27]. However, the effectiveness of antibiotic treatment relies on the accurate identification of causative pathogens. To confirm the diagnosis of osteomyelitis and accurately determine the responsible microorganisms, bone biopsy culture is considered the gold standard. However, the invasive nature of bone biopsy, as well as the technical challenges, and the risk of complications limit its integration into routine clinical practice [28,29].

As a less invasive and more practical alternative, deep tissue cultures are frequently used in clinical practice. Slater et al. emphasized that, due to the limitations of bone biopsy, there is an increasing reliance on soft tissue cultures in clinical settings [30]. However, the extent to which deep tissue cultures accurately represent osteomyelitis pathogens and their diagnostic reliability remains controversial. In particular, determining the diagnostic accuracy of deep tissue cultures becomes crucial in cases where bone biopsy is not feasible. In this context, studies evaluating the diagnostic reliability of deep tissue cultures are becoming increasingly important for establishing the optimal treatment strategies in the management of DFO.

Studies evaluating the diagnostic concordance between bone biopsy cultures, which are considered the gold standard for osteomyelitis diagnosis, and other tissue cultures have reported varying concordance rates. Senneville et al. found a 22.5% concordance between superficial swab cultures and percutaneous bone biopsy cultures, demonstrating that superficial swabs are not a reliable method for diagnosing osteomyelitis [31]. A prospective study from India, which included 144 patients, reported a concordance rate of only 38.2% between bone biopsy and superficial swab cultures [32]. Liu et al. found a 42.8% concordance between deep tissue and bone biopsy cultures [33]. In our study, the overall concordance between deep tissue and bone biopsy cultures was 51.8%, which is higher than most previously reported findings. Additionally, Cohen’s kappa coefficient was calculated to assess the concordance between the two sampling methods beyond chance, yielding a value of 0.48, which reflects a moderate level of concordance. Similarly, Ertugrul et al. reported a moderate level of concordance between deep tissue and bone cultures in their prospective study [34]. By contrast, a 2020 systematic review reported lower concordance rates, ranging from 19% to 42%, with the highest rates observed for *S. aureus* [35].

These findings indicate that deep tissue cultures should not be completely disregarded; however, they do not serve as a full alternative to a bone biopsy. Nevertheless, there are studies supporting the reliability of deep tissue cultures. Malone et al. reported a concordance rate of 73.5% between deep tissue cultures and either surgical or percutaneous bone biopsies and suggested that deep tissue cultures should not be overlooked, particularly in centers where bone biopsy is not feasible [36].

In the literature, some studies have proposed sinus tract cultures as an alternative diagnostic method for osteomyelitis. A non-randomized, prospective study from Switzerland, which included 54 cases of osteomyelitis, demonstrated that two consecutive sinus tract cultures could accurately predict the causative pathogen in monomicrobial infections [37]. Similarly, in a cross-sectional, prospectively designed study by Soomro et al., which included 90 patients with chronic osteomyelitis, it was suggested that sinus tract cultures might provide valuable microbiological information when interpreted cautiously. However, the study also emphasized that sinus tract cultures carry a high risk of contamination and should therefore be carefully evaluated in clinical decision-making [38].

In our study, *S. aureus* was identified as the most frequently isolated pathogen in both bone biopsy and deep tissue cultures. This finding is consistent with the study conducted by Hartemann-Heurtier and Senneville (2008) and supports the notion that *S. aureus* is one of the primary causative pathogens in DFO [39]. Additionally, the highest concordance rate between bone biopsy and deep tissue cultures was observed in cases with *S. aureus* isolation (44.4%). Notably, the most recent guidelines of the IWGDF emphasize that, in cases where a single virulent pathogen, particularly *S. aureus*, is isolated from an aseptically obtained deep soft tissue sample, a bone biopsy may not be necessary [40]. This recommendation aligns with the findings of our study, suggesting that deep tissue cultures may hold diagnostic value in cases dominated by *S. aureus*. However, bone biopsy remains essential for the identification of other pathogens, particularly in polymicrobial infections, where advanced diagnostic techniques may be more appropriate.

When the pathogens isolated from deep tissue and bone cultures of the patients included in our study were classified as Gram-positive and Gram-negative microorganisms, Gram-negative microorganisms were found to be more frequently isolated. The most commonly detected Gram-negative bacteria were *E. coli* and *K. pneumoniae.* This finding suggests that Gram-negative bacteria play a significant role in the pathogenesis of DFOs and should be considered when selecting empirical antibiotic therapy. However, a major concern emerged in the evaluation of culture results for Gram-negative bacteria: the concordance rate between bone biopsy and deep tissue cultures for Gram-negative organisms was only 31.9%. This rate is markedly lower than the concordance observed for *S. aureus* and other Gram-positive organisms. Gram-negative bacteria are often associated with more resistant, invasive, and difficult-to-treat infections. Therefore, accurate identification of the causative pathogen is crucial for effective patient management. The limited ability of deep tissue cultures to accurately detect Gram-negative pathogens may result in inappropriate or suboptimal antibiotic therapy, potentially leading to clinical failure. Similarly, a study conducted by Pellizzer et al. reported that wound cultures frequently fail to isolate Gram-negative organisms, which may contribute to inadequate treatment strategies and poorer outcomes [41]. In this context, when Gram-negative infections are suspected or confirmed, performing a bone biopsy may become essential to improve diagnostic accuracy and therapeutic effectiveness. Bone biopsy plays a particularly critical role in cases of polymicrobial infections, chronic or recurrent osteomyelitis, poor clinical response to empirical antibiotics, or a known history of multidrug-resistant Gram-negative organisms. While deep tissue cultures may be sufficient in selected cases such as monomicrobial *S. aureus* infections, bone biopsy remains an indispensable diagnostic tool in more complex, resistant, or multi-organism infections.

In our study, 21.2% of patients had positive deep tissue cultures but negative bone cultures, whereas 16.5% had positive bone cultures but negative deep tissue cultures. This discrepancy may be attributed to the heterogeneous distribution of infection within anatomical compartments or the effects of prior antibiotic exposure.

Antibiotic therapy can significantly affect culture outcomes. Prior studies have shown that previous antibiotic use may lead to false-negative culture results in both bone and deep tissue samples, and discontinuing antibiotics before biopsy increases the likelihood of pathogen detection [42,43]. However, the optimal antibiotic-free interval before biopsy remains uncertain, with some experts recommending a minimum of several days, ideally two weeks, before obtaining bone biopsy specimens [43]. In our study, the majority of patients (96.3%) had a history of systemic antibiotic use within the preceding three months; however, none were receiving antibiotics at the time of specimen collection. The fact that all patients had discontinued antibiotics for at least 15 days prior to culture sampling can be considered favorable in terms of improving culture sensitivity. Nevertheless, considering that some patients had received antibiotics for durations extending to eight weeks, the impact of prior antimicrobial exposure on culture results cannot be excluded. Previous antibiotic therapy may suppress viable pathogens, alter the microbial flora, or lead to the selection of more resistant and fastidious organisms, thereby reducing culture yield. Fastidious pathogens are microorganisms that require specific nutritional or environmental conditions for growth and are difficult to isolate using standard laboratory culture techniques. Examples include anaerobes, Actinomyces spp., and certain slow-growing bacteria. These effects may have contributed to the culture-negative or microbiologically discordant findings observed in some cases in our study.

Although patients were not receiving antibiotics at the time of sampling, the retrospective design of this study limits our ability to fully assess the precise impact of prior antimicrobial therapy on culture outcomes. This represents one of the major limitations of our study.

In our study, anaerobic bacteria were not isolated, likely due to the limitations in anaerobic bacterial culture conditions in our hospital laboratory. Despite numerous studies on DFI over the past two decades, the true prevalence of anaerobic pathogens in DFI remains uncertain. This uncertainty is largely attributed to the lack of standardization in bacterial culture methods across studies. Factors such as the type of sample collected for analysis, the conditions and efficiency of sample transport to the microbiology laboratory, and the processing methods used significantly influence the detection rate of anaerobic bacteria [44]. In our study, no bacterial growth was observed in 39.3% of the deep tissue cultures and in 33.3% of the bone cultures. Considering the high prevalence of peripheral arterial occlusion in our patient cohort, it is plausible that anaerobic bacteria could be the causative pathogens in these culture-negative cases. If anaerobic bacteria had been successfully cultured, the concordance rate between bone and tissue cultures might have been higher. In this context, replacing conventional culture techniques with advanced molecular diagnostic methods could enhance the identification of anaerobic bacteria in the etiology of DFI, providing a more accurate assessment of their role [45].

Traditional culture methods may be insufficient for detecting anaerobic bacteria and fastidious pathogens. In recent years, molecular techniques such as 16S rRNA gene sequencing and metagenomic next-generation sequencing (mNGS) have emerged as promising tools with the potential to identify DFO pathogens with higher sensitivity [45,46]. These methods offer several advantages, including the ability to detect pathogens even in culture-negative cases, improved identification of polymicrobial infections, and the potential for personalized antimicrobial therapy. However, high costs, the need for specialized laboratory infrastructure, uncertainties in clinical interpretation, and the inability to distinguish between viable and non-viable bacteria remain significant limitations to their routine use [46,47,48]. Future studies should focus on evaluating the correlation between these tests and clinical outcomes, assessing the impact of combining them with conventional culture methods on diagnostic accuracy, and conducting cost-effectiveness analyses to determine their feasibility for routine clinical implementation. In this context, integrating advanced molecular diagnostic techniques into routine clinical practice could substantially improve diagnostic accuracy, particularly in cases where conventional cultures are insufficient or polymicrobial infections are suspected. These technologies could initially be incorporated into diagnostic algorithms at tertiary or academic medical centers, especially for culture-negative or clinically complex cases. Pilot studies are needed to compare these methods with conventional cultures in terms of diagnostic accuracy, turnaround time, and impact on antimicrobial therapy. Additionally, cost-effectiveness analyses and the standardization of protocols for sample collection, processing, and reporting will be essential for broader clinical adoption. Future research should focus on evaluating the clinical effectiveness of molecular-guided therapy, long-term patient outcomes, and the feasibility of incorporating these technologies into routine care pathways.

This study has some important limitations. First, its retrospective design may have introduced selection bias and confounding factors. Second, our patient population was limited to a single center, restricting the generalizability of microbiological findings to other geographic regions. Third, only conventional culture methods were used, without incorporating molecular diagnostic approaches, potentially underestimating the presence of anaerobic or difficult-to-culture microorganisms. Lastly, while we assessed microbiological concordance, we did not evaluate the direct impact of culture-based diagnostic strategies on patient outcomes in a prospective manner, which limits our ability to correlate diagnostic accuracy with clinical efficacy. Moreover, because the primary aim of this study was diagnostic, treatment strategies and clinical outcomes were not systematically evaluated and were considered beyond the scope of this investigation.

The most recent guidelines of the IWGDF [40] continue to recommend bone biopsy as the preferred diagnostic method for DFO. In the future, the integration of advanced imaging techniques and novel microbiological diagnostic methods into clinical practice may provide less invasive and more practical alternatives for the diagnosis of osteomyelitis. However, the reliability of these methods must be validated, and their standardization for clinical use remains essential. At present, bone biopsy continues to be regarded as the gold standard, particularly for culture-based diagnosis.

## 5. Conclusions

This study demonstrates that the microbiological concordance between deep tissue and bone biopsy cultures in the diagnosis of diabetic foot osteomyelitis remains limited. The concordance rate of 51.8% suggests that deep tissue cultures may provide guidance in clinical decision-making, particularly in cases with *S. aureus* isolation. However, the low concordance rates observed for Gram-negative bacteria and other pathogens indicate that deep tissue cultures alone may not be sufficient for the diagnosis of osteomyelitis. Therefore, there remains a need for diagnostic methods that provide reliability comparable to bone biopsy cultures while being less invasive and more easily integrated into routine clinical practice. Although novel molecular diagnostic techniques and advanced imaging modalities have the potential to improve the diagnostic process of DFO, further studies are required to validate their effectiveness and clinical utility.

Based on our findings, deep tissue cultures may be considered sufficient for guiding antimicrobial therapy in select cases with monomicrobial *S. aureus* infection confirmed by aseptic sampling. However, bone biopsy remains indispensable in patients with polymicrobial infections, recurrent or non-healing ulcers, prior antibiotic failure, or suspected multidrug-resistant organisms.

In conclusion, further research is necessary to develop diagnostic strategies that enhance accuracy, improve clinical applicability, and optimize patient management in DFO. Specifically, future studies should focus on determining the diagnostic reliability of deep tissue cultures in different patient populations, comparing the efficacy of molecular and advanced diagnostic approaches with bone biopsy, and evaluating their impact on clinical outcomes.

## Figures and Tables

**Table 1:** Patient characteristics.

Age, mean ± SD *	63.449 ± 12.363
Sex (male), *n* (%)	80 (74.8%)
Any comorbid disease, *n* (%)	
Diabetes Mellitus	107 (100%)
Hypertension	100 (93.5%)
Peripheral neuropathy	98 (91.5%)
Peripheral vascular obstruction	96 (89.7%)
Atherosclerosis	81 (75.7%)
Congestive heart failure	31 (29%)
Chronic renal failure	26 (24.3%)
Venous stasis	4 (3.7%)
Chronic obstructive pulmonary disease	1 (0.9%)
Cerebrovascular accident	1 (0.9%)
Hepatitis	1 (0.9%)
Lymphedema	1 (0.9%)
Previous antibiotic use	103 (96.3%)

* Abbreviation: SD = standard deviation.

**Table 2:** Grade of ulcerations in the study population.

IDSA/IWGDF Classification	No (%)
2	5 (4.7)
3	69 (64.5)
4	33 (30.8)

**Table 3:** Pathogens identified in deep tissue and bone biopsy cultures of patients.

Pathogen	Deep Tissue Cultures (*n* = 107), *n* (%)	Bone Cultures (*n* = 105), *n* (%)
*Staphylococcus aureus*	14 (13.1)	13 (12.2)
MRSA	6 (5.6)	7 (6.7)
*Escherichia coli*	9 (8.4)	7 (6.5)
*Klebsiella pneumoniae*	5 (4.7)	9 (8.4)
*Proteus* spp.	5 (4.7)	6 (5.6)
*Pseudomonas aeruginosa*	5 (4.7)	5 (4.7)
*Corynebacterium striatum*	4 (3.7)	6 (5.6)
*Morganella morganii*	3 (2.8)	2 (1.9)
*Enterococcus faecalis*	3 (2.8)	–
*Acinetobacter baumannii*	3 (2.8)	–
*Streptococcus* spp.	3 (2.8)	8 (7.5)
*Coagulase-negative staphylococci*	2 (1.9)	5 (4.7)
*Citrobacter* spp.	2 (1.9)	3 (2.8)
*Enterobacter cloacae*	2 (1.9)	1 (0.9)
*Achromobacter*	1 (0.9)	–
*Helcococcus kunzii*	1 (0.9)	1 (0.9)
*Providencia*	1 (0.9)	2 (1.9)
*Serratia marcescens*	1 (0.9)	–
*Candida* spp.	1 (0.9)	1 (0.9)
*Ralstonia picketti*	–	1 (0.9)
No growth	42 (39.3)	35 (33.3)
Total	107 (100)	105 (100)

**Table 4:** Concordance of deep tissue and bone biopsy cultures, *n* (%).

Same microorganism isolated	44 (51.8)
Different microorganisms isolated	9 (10.6)
Deep Tissue culture only	18 (21.2)
Bone culture only	14 (16.5)

**Table 5:** Concordance between deep tissue and bone sample cultures according to microorganism type.

	Total	Deep Tissue	Bone Biopsy	Concordance, *n* (%)
*S. aureus*	27	14	13	12 (44.4)
Other Gram-positive	33	13	20	8 (24.2)
Gram-negative	73	37	36	23 (31.5)

## Data Availability

All data needed to support the conclusions are present in the paper. Raw data are available from the corresponding author, S.U., upon reasonable request.
